# Supplementary material for: Cationic Copolymerization of Isobutylene with 4-Vinylbenzenecyclobutylene: Characteristics and Mechanisms
Source: Polymers (Basel). 2020 Jan 13;12(1):201. doi: 10.3390/polym12010201 (PMC7023408; doi:10.3390/polym12010201)
Supplement: Supplementary file 1 [file polymers-12-00201-s001.pdf]

Supporting Information

Title: Cationic Copolymerization of Isobutylene with 4-vinylbenzenecyclobutylene: Characteristics and Mechanisms

Corresponding Author: **Yibo Wu**

Authors: Zhifei Chen, Shuxin Li*, Yuwei Shang, Shan Huang, Kangda Wu, Wenli Guo and Yibo Wu*

*To whom correspondence should be addressed.

Tel.: 13810375915

E-mail: wuyibo@bipt.edu.cn

1. Living Characteristics of Polymerization of Isobutylene

In this experiment, polymerization is performed in a glove box under dry nitrogen atmosphere, we used TMPCl as the initiator and $TiCl_4$ as the coinitiator. We also added the proton trap DTBP to stabilize the cationic active center. Take a typical polymerization process as an example: 15 ml of cyclohexane, 10 ml of CH_3Cl, $3.28×10^{-4}$ mol of TMPCl, $8.48×10^{-4}$ mol of DTBP, and 0.01 mol of $TiCl_4$ were added sequentially to a polymer bottle which had been drained of water, the reagents were homogenized and hold at -80℃ for 30 min, then 0.03 mol of IB was added and polymerization was starting. After the reaction went on a certain time, the polymerization was quenched with 10 ml prechilled ethanol. The polymer products were dried in a vacuum oven at 40 °C to a constant weight overnight. The monomer conversion was determined gravimetrically. (1.59g).

Table 1. Polymers of Isobutylene with different reaction time initiated by TMPCl/DTBP/$TiCl_4$ system, [TMPCl] = $2.87×10^{-3}$ M, [DTBP] = $7.18×10^{-3}$ M, [$TiCl_4$] = 0.09 M, and [IB] = 1.01 M in cyclohexane /CH_3Cl (60/40 v/v) at -80 °C.

Time(min)	Yiled(%)	$M_n(g \cdot mol^{-1})$	M_W/M_N
20	9	4370	1.23
30	19	8150	1.31
45	45	20700	1.25
60	74	39200	1.35
90	88	43600	1.23

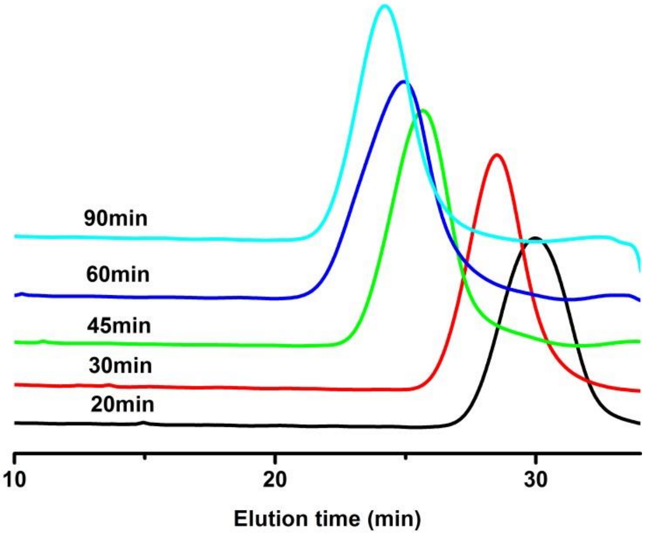

Figure 1. GPC RI traces of polymerization of IB initiated by TMPCl/DTBP/$TiCl_4$ system in cyclohexane/CH_3Cl (60/40 v/v) at -80 °C., [TMPCl] = $2.87×10^{-3}$ M, [DTBP] = $7.18×10^{-3}$ M, [TiCl4] = 0.09 M, and [IB] = 1.01 M.

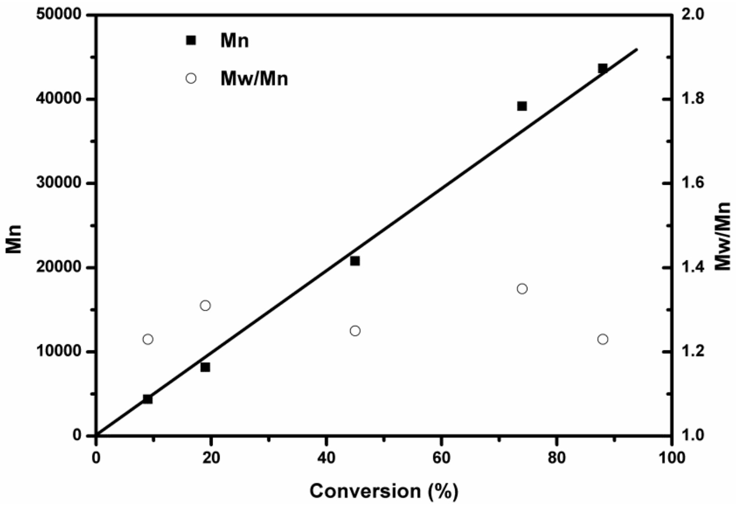

Figure 2. *Mn* and *Mw*/*Mn* as a function of conversion for the polymerization of IB initiated by TMPCl/DTBP/$TiCl_4$ system in cyclohexane/CH_3Cl (60/40 v/v) at -80 °C., [TMPCl] = 2.87×10^{-3} M, [DTBP]=7.18×10^{-3} M, [$TiCl_4$]=0.09M and [IB]=1.01M.

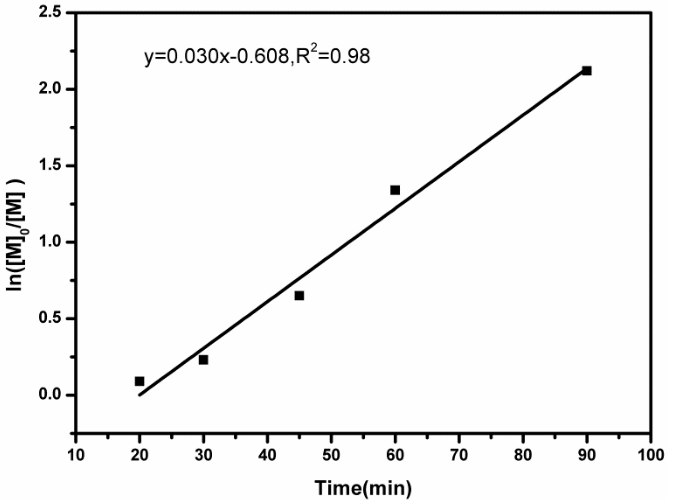

Figure 3. First-order plot for the polymerization of IB initiated by TMPCl/DTBP/$TiCl_4$ system in cyclohexane/CH_3Cl (60/40 v/v) at -80 ℃, [TMPCl] = 2.87×10^{-3} M, [DtBp] = 7.18×10^{-3} M, [TiCl4] = 0.09 M and [IB] = 1.01 M.

We designed several polymerization experiments with different reaction times (Table1). Weight differences method were used to determine the conversion of IB monomer (conversion $= \frac{m_{0}-m_{t}}{m_0} \times 100\%$, in which m_0 is the starting weight of IB, and m_t is the weight of IB had been polymerized for a predesigned time). Figure 1 shows the GPC traces of the PIB obtained at different polymerization time. As the polymerization time increases, the peak position of PIB moves towards higher molecular weight. Figure 2 shows the relationship between *Mn* and PID of PIB with different polymerization times and the conversion of monomer. It indicated that *Mn* and monomer conversion have a linear growth relationship, and PDI with different polymerization times remains narrow. Figure 3 shows the curve of $\ln [M]_0/[M]_t$ with reaction time, where $[M]_0$ is the starting monomer concentration and $[M]_t$ is the monomer concentration after a certain polymerization time, and there is a first order

linear relationship between the both. These characteristics indicate that there were few chain termination and transfer reactions in the polymerization process of IB, and these properties are consistent with the characteristics of living polymerization.

2. Derivation of the equation for the calculation of monomer reactivity ratio

When calculating the monomer reactivity ratio, we controlled the conversion rate below 15%. At this stage the copolymer composition can be assumed to be constant. There are four types of chain growth:

$$[\mathrm{IB}\cdot] + [\mathrm{IB}] \xrightarrow{k_{11}} \sim\mathrm{IB}\cdot$$

$$[\mathrm{IB}\cdot] + [4-\mathrm{VBCB}] \xrightarrow{k_{12}} \sim 4-\mathrm{VBCB}\cdot$$

$$[4-\mathrm{VBCB}\cdot] + [\mathrm{IB}] \xrightarrow{k_{21}} \sim\mathrm{IB}\cdot$$

$$[4-\mathrm{VBCB}\cdot] + [4-\mathrm{VBCB}] \xrightarrow{k_{22}} \sim 4-\mathrm{VBCB}\cdot$$

Where, $[\mathrm{IB}\cdot]$ and $[4-\mathrm{VBCB}\cdot]$ represent the concentration of the active chain with terminal IB and 4-VBCB, and $[\mathrm{IB}]$ and $[4-\mathrm{VBCB}]$ represent the concentration of monomer IB and 4-VBCB, respectively. When the chain growth rate is stable, the concentration of two active centers should be constant:

$$k_{21}[\mathrm{IB}\cdot][4-\mathrm{VBCB}] = k_{22}[4-\mathrm{VBCB}\cdot][\mathrm{IB}] \quad (1)$$

The rate at which IB and 4-VBCB polymerize to the copolymer chain is:

$$\frac{d[\mathrm{IB}]}{dt} = k_{11}[\mathrm{IB}\cdot][\mathrm{IB}] + k_{21}[4-\mathrm{VBCB}\cdot][\mathrm{IB}] \quad (2)$$

$$\frac{d[4-\mathrm{VBCB}]}{dt} = k_{12}[\mathrm{IB}\cdot][4-\mathrm{VBCB}] + k_{22}[4-\mathrm{VBCB}\cdot][4-\mathrm{VBCB}] \quad (3)$$

From formula 2 and 3, we get:

$$\frac{D[\mathrm{IB}]}{d[4-\mathrm{VBCB}]} = \frac{k_{11}[\mathrm{IB}\cdot][\mathrm{IB}] + k_{21}[4-\mathrm{VBCB}\cdot][\mathrm{IB}]}{k_{12}[\mathrm{IB}\cdot][4-\mathrm{VBCB}] + k_{22}[4-\mathrm{VBCB}\cdot][4-\mathrm{VBCB}]} \quad (4)$$

$$\frac{d[\mathrm{IB}]}{d[4-\mathrm{VBCB}]} = \frac{k_{11}[\mathrm{IB}]/k_{12}[4-\mathrm{VBCB}] + 1}{1 + k_{22}[4-\mathrm{VBCB}]/k_{21}[\mathrm{IB}]} = \frac{[\mathrm{IB}]}{[4-\mathrm{VBCB}]} \times \frac{(k_{11}/k_{12})[\mathrm{IB}] + [4-\mathrm{VBCB}]}{(k_{22}/k_{21})[4-\mathrm{VBCB}] + [\mathrm{IB}]} = = \frac{[\mathrm{IB}]}{[4-\mathrm{VBCB}]} \times \frac{r_1[\mathrm{IB}] + [4-\mathrm{VBCB}]}{r_2[4-\mathrm{VBCB}] + [\mathrm{IB}]} \quad (5)$$

Let,

$$x = \frac{[\mathrm{IB}]}{[4-\mathrm{VBCB}]}$$

$$y = \frac{d[\mathrm{IB}]}{d[4-\mathrm{VBCB}]}$$

If the conversion rate is less than 15%, x is treated as the starting molar ratio of IB to 4-VBCB; y

is treated as the molar ratio of IB to 4-VBCB in the copolymer. Substitute x and y into equation (5),

$$y = \frac{r_1 x + 1}{1 + r_2/x}$$

$$\left(\frac{x}{y^{1/2}}\right) r_1 - \left(\frac{y^{1/2}}{x}\right) r_2 + \left(\frac{1}{y^{1/2}} - y^{1/2}\right) = 0 \tag{6}$$

Process Equation (6) as least squares：

$$r_1 A_1 - r_2 n = C_1$$

$$-r_1 n + r_2 A_2 = C_2$$

$$r_1 = \frac{A_2 C_1 + n C_2}{A_1 A_2 - n^2}$$

$$r_2 = \frac{A_1 C_2 + n C_1}{A_1 A_2 - n^2}$$

Where, $A_1 = \sum_{i=1}^{n} \frac{x_i^2}{y_i}$, $A_2 = \sum_{i=1}^{n} \frac{y_i}{x_i^2}$, $C_1 = \sum_{i=1}^{n} x_i \left(1 - \frac{1}{y_i}\right)$, $C_2 = \sum_{i=1}^{n} \frac{y_i}{x_i}\left(\frac{1}{y_i} - 1\right)$, n=5.
